# Effects of Cadence Control on Upper-Limb Kinematics and Muscle Activation During Manual Wheelchair Propulsion in Individuals with Spinal Cord Injury

**DOI:** 10.3390/life15121885

**Published:** 2025-12-10

**Authors:** Soonbeom Kim, Jiyoung Park, Seon-Deok Eun, Dongheon Kang

**Affiliations:** 1 Clinical Rehabilitation Research, National Rehabilitation Center, Seoul 01022, Republic of Korea; ksb0406@korea.kr; 2 Department of Safety and Health, Wonkwang University, Iksan 54538, Republic of Korea; withji0@wku.ac.kr; 3 Assistive Technology Research Team for Independent Living, National Rehabilitation Center, Ministry of Health and Welfare, Seoul 01022, Republic of Korea

**Keywords:** spinal cord injury, manual wheelchair, propulsion, biomechanics, electromyography

## Abstract

Manual wheelchair propulsion is a frequent activity among people with spinal cord injury (SCI) and is linked to upper limb loading and shoulder pain. We compared propulsion strategies at cadences of 30 and 50 bpm. Kinematics and surface electromyography (EMG) were recorded across the propulsion cycle, push/recovery phases, and events. Ranges of motion for shoulder flexion/extension, adduction/abduction, and elbow flexion/extension did not differ significantly, although ROM tended to be smaller at 50 bpm; push angle was larger at 50 bpm but not significant. Propulsion cycle duration was shorter at 50 bpm (*p* < 0.001). Push duration was similar, but its proportion of the cycle increased at 50 bpm (*p* < 0.001). Recovery duration was shorter at 50 bpm (*p* < 0.001), yet its cycle proportion increased (*p* < 0.01). EMG showed cadence-specific redistribution: higher activity at 50 bpm at preparation (anterior deltoid, pectoralis major, biceps brachii, upper trapezius; *p* < 0.01) and at contact (posterior deltoid; *p* < 0.05); higher biceps brachii at release and higher anterior deltoid at end-range extension at 30 bpm (both *p* < 0.05). Cadence manipulation reorganized timing and muscle demands without large ROM changes, supporting rhythm-based training and propulsion design to mitigate shoulder loading.

## 1. Introduction

Manual wheelchairs are widely preferred mobility devices for individuals with physical impairment because they are low-cost and require minimal maintenance [1]. Among people with spinal cord injury (SCI), manual wheelchair propulsion is one of the most frequent daily activities [2]. Recent reports indicate a high prevalence of shoulder pain in manual wheelchair users, and interest is growing in the relationship between propulsion technique and shoulder loading.

Manual wheelchair propulsion involves repetitive movements and is associated with an increased risk of upper-limb pain and injury [3]. Repetition propulsion motion imposes cumulative loads on the shoulder, elbow, and wrist, compounded by a high frequency of push strokes, thereby causing pain and functional limitations [4]. The manual wheelchair propulsion is generally characterized by low mechanical efficiency and substantial upper-limb loading, which elevates the risk of musculoskeletal pathology—such as shoulder impingement—and can adversely affect independence and quality of life. Consistent with this, prior studies describe manual wheelchair use as a physically demanding and relatively inefficient mode of mobility, with heightened risk of upper-extremity pain and pathology that often leads to reduced functional independence and diminished quality of life [5,6,7].

Understanding upper extremity joint patterns is crucial for preventing injuries in manual wheelchair users, including those with SCI. In particular, propulsion strategy analysis is a key component of rehabilitation for individuals with SCI [8]. Research on wheelchair design and biomechanics is essential to understanding propulsion mechanisms, how external and internal features of the musculoskeletal system interact, and strategies for maintaining the user’s joints [9]. Recently, there has been a rapid increase in interest in various elements of manual wheelchair propulsion methods to reduce the strain on upper extremity joints and muscles in manual wheelchair users and individuals with SCI. Knowledge of wheelchair propulsion data and trends can provide additional information for activity planning to prevent overuse injuries or for analyzing the outcomes of health-related interventions in the areas of activity and participation [10,11,12].

Nevertheless, prior studies on manual wheelchair propulsion have several limitations. First, many investigations have focused on comparisons at self-selected speed or across different speed conditions, and only a limited number have consistently evaluated the effects under a fixed cadence/tempo, specifically on upper-limb kinematics and muscle-activation patterns. Second, relatively few studies have simultaneously measured changes in joint angles and range of motion (ROM) together with the distribution/timing of muscle activation as propulsion speed and cadence vary.

For this reason, this study has the following purposes. The purpose of this study is to analyze the differences and pattern changes between the kinematic strategy and muscle activity of the upper limb when maintaining a constant speed in patients with SCI, while controlling the beat at two conditions: 30 bpm and 50 bpm. Through this, it is intended to verify whether biomechanical variables change significantly when only the propulsion rhythm is controlled. And it is intended to provide the basis for the design of an efficient and low-risk propulsion strategy.

## 2. Materials and Methods

### 2.1. Participants

Eighteen adults with SCI were voluntarily recruited from rehabilitation centers and community welfare facilities in Seoul, Republic of Korea. The inclusion criteria were as follows: (1) adults aged 19 years or older with a diagnosis of spinal cord injury for at least 12 months, (2) individuals with paraplegia who use a manual wheelchair for mobility in daily life, and (3) individuals who do not engage in professional sports activities. The exclusion criteria were as follows: (1) individuals with clinically significant mental or cognitive impairment, (2) individuals who are unable to understand the explanation of the study or follow the investigator’s instructions, and (3) individuals with a history of musculoskeletal injury to the upper extremities. All participants were male (mean age, 60.89 ± 8.92 years); mean height was 168.86 ± 7.10 cm, body mass 66.64 ± 17.12 kg, and time since injury 18.06 ± 8.70 years. Lesion levels were T7–T12 in 14 participants and L1 or L3 in 4 participants. General characteristics are summarized in Table 1. Individuals were excluded if they had musculoskeletal disorders or a history of fracture/dislocation that could affect wheelchair propulsion. The study was approved by the Institutional Review Board of the National Rehabilitation Center, Seoul, Republic of Korea (IRB No.: NRC-2024-04-018), and all participants provided written informed consent. The study adhered to the principles of the Declaration of Helsinki.

### 2.2. Experimental Procedure and Data Acquisition

Upper-limb kinematics during manual wheelchair propulsion were recorded using a 10-camera motion-capture system (Vicon Motion Systems, Oxford, UK) at 100 Hz. Further, 14-mm retroreflective markers were placed according to a modified Plug-in Gait scheme to track upper-limb segments. To assess muscle activation and timing, a wireless surface EMG system (Trigno, Delsys, Inc., Boston, MA, USA) was used to record seven muscles—anterior deltoid (AD), middle deltoid (MD), posterior deltoid (PD), biceps brachii (BB), triceps brachii (TB), pectoralis major (PM), and upper trapezius (UT)—on the dominant limb, following SENIAM recommendations. EMG data were sampled at 2000 Hz.

**Figure 1:**
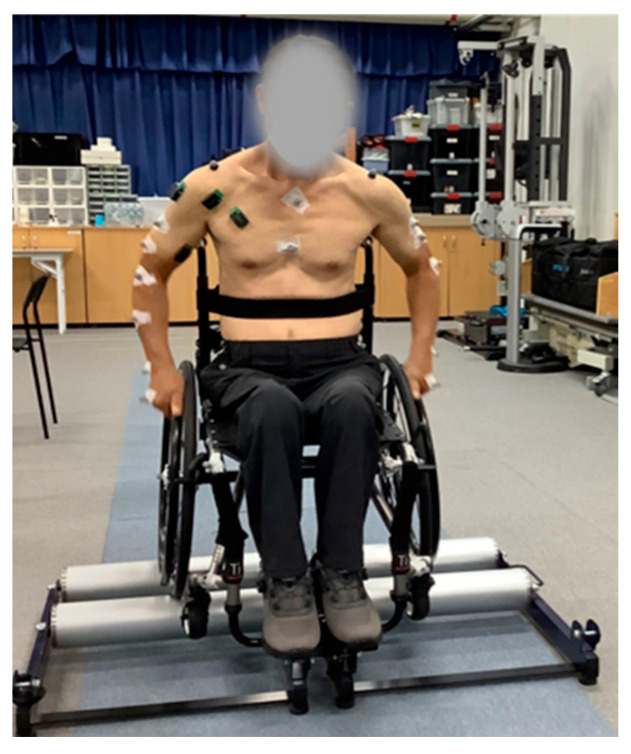
Placement of reflective markers and sEMG sensor, and setup for wheelchair propulsion assessment.

After marker and electrode placement, participants propelled an active manual wheelchair (TiSport TRA, Pasco, WA, USA) on a wheelchair roller ergometer (WT-380, Fitcare Co., Ltd., Seoul, Republic of Korea) (Figure 1). To cue the two cadence conditions, a smartphone metronome application (Metronome: Tempo, Frozen Ape Pte. Ltd., Singapore) was used. Participants were provided with sufficient familiarization with both the ergometer and the metronome prior to testing. Participants performed wheelchair propulsion assessments under two metronome-paced cadence conditions, 30 and 50 bpm, completing five trials in each condition. The 30 bpm condition was defined as a low-speed cadence with reference to the study by Kukla et al. [13], which controlled push frequency during manual wheelchair propulsion using a metronome set at 30 bpm. The 50 bpm condition was selected as a moderate cadence representative of typical everyday propulsion speeds, considering the range of push frequencies reported in previous wheelchair propulsion studies, including the 60 bpm metronome condition used by Bickelhaupt et al. [14]. Testing was terminated immediately if any pain or discomfort was reported to ensure the participant’s safety.

### 2.3. Data Processing

**Figure 2:**
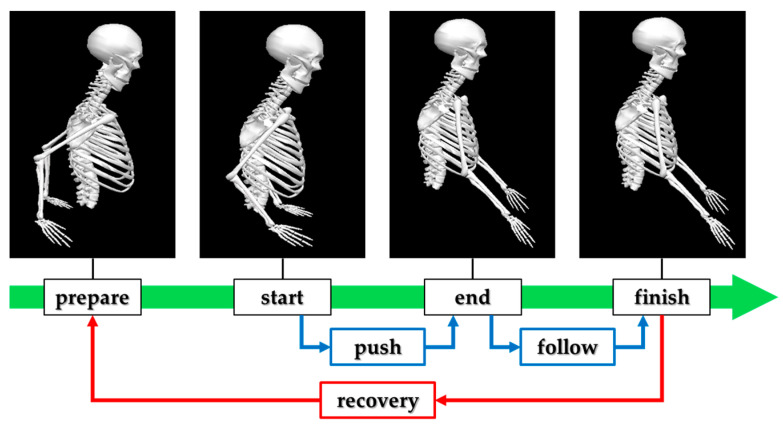
Definition of event and phase during manual wheelchair propulsion.

Upper-limb kinematics and surface EMG were processed using Visual 3D v6 Professional (HAS-Motion Inc., Kingston, ON, Canada; formerly C-Motion Inc., Germantown, MD, USA). An upper-limb model was created for each participant based on the recorded marker set. Before joint/segment analysis, marker trajectories were low-pass filtered with a fourth-order Butterworth filter at 6 Hz to reduce noise. Joint angles of the upper limb were then computed relative to the segment coordinate systems using an X–Y–Z Cardan sequence. For between-condition comparisons, task events and phases of the wheelchair propulsion cycle were defined as in Figure 2: prepare (the instant when the hand is at its most posterior position), start (initial hand–rim contact initiating the push), end (hand–rim release at the end of the push), and finish (the instant of maximal forward reach after push). The push phase was defined from start to end, the follow phase from end to finish, and the recovery phase from finish to prepare.

**Figure 3:**
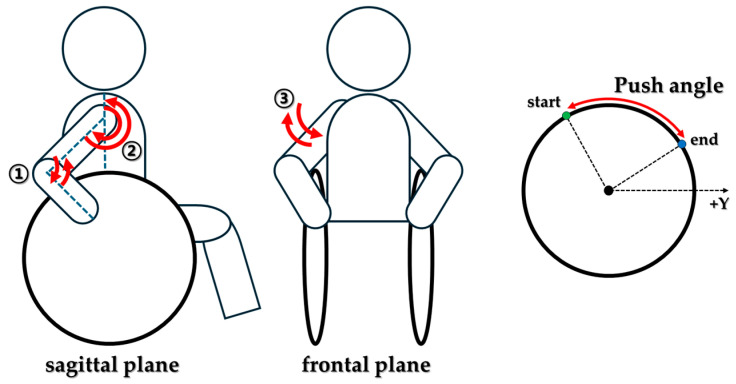
Define the Upper extremity joint angle and wheelchair push angle.

The joint angles used for upper-limb kinematic comparisons were defined as in Figure 3:

(1) Shoulder flexion/extension—the relative angle between the thorax and humerus in the sagittal plane; and
(2) Elbow flexion/extension—the relative angle between the humerus and forearm in the sagittal plane; and
(3) Shoulder abduction/adduction—the relative angle between the thorax and humerus in the frontal plane.

To characterize propulsion technique, the push angle was computed as the handrim sweep angle during the push phase, i.e., the angular displacement of the handrim contact point from initial contact (start) to release (end) with respect to the wheel center.

To analyze muscle activation patterns under the two cadence conditions, raw surface EMG signals were band-pass filtered using a fourth-order Butterworth filter (20–450 Hz) to remove noise. Signals were then full-wave rectified and smoothed with a 100 ms RMS window. For between-condition comparison, amplitudes were normalized using the reference voluntary contraction (RVC) method, where RVC was defined as the EMG level recorded. At the same time, the participant was seated in a comfortable, upright posture. To compare muscle activity across propulsion phases, integrated EMG (iEMG) was computed within each phase (push and recovery), and event-based amplitudes were extracted at the prepare, start, end, and follow events. All kinematic and sEMG analyses were performed on the dominant upper limb only. The dominant side was determined by identifying the participant’s dominant side.

### 2.4. Statistical Analysis

Kinematic and EMG data were analyzed using SPSS (Version 21.0; IBM Corp., Armonk, NY, USA). Participant characteristics were summarized using descriptive statistics (mean ± SD). Normality of continuous variables was assessed with the Shapiro–Wilk test; because the assumption of normality was violated (*p* < 0.05), between-cadence comparisons (30 vs. 50 bpm) were conducted with the Wilcoxon signed-rank test. The statistical significance level was set at *p* < 0.05.

## 3. Results

The results of the comparative analysis of upper limb kinematics and muscle activity for the two propulsion conditions are as follows.

### 3.1. Kinematics

The comparative results for upper-limb kinematics under the two propulsion conditions are presented in Table 2. Analyses of the ranges of motion (ROM) for elbow flexion/extension, shoulder flexion/extension, and shoulder adduction/abduction across the propulsion cycle, push phase, and recovery phase showed no significant difference between 30 bpm and 50 bpm. Overall, ROM values for all upper-limb joints tended to be smaller at 50 bpm. In particular, elbow ROM during the recovery phase showed the most significant absolute difference (30 bpm: 60.45 ± 28.65°; 50 bpm: 50.20 ± 14.00°). The push angle—the angular sweep on the handrim while the hand is in contact and generating propulsion—was relatively larger at 50 bpm, but the difference was not statistically significant.

For temporal variables of wheelchair propulsion, propulsion-cycle duration was 2.07 ± 0.25 s at 30 bpm and 1.29 ± 0.37 s at 50 bpm, being significantly shorter at 50 bpm (*p* < 0.001). Push-phase duration did not differ significantly between 30 and 50 bpm; however, the push-phase proportion of the cycle was 15.93 ± 2.18% at 30 bpm and 24.93 ± 3.05% at 50 bpm, which was significantly greater at 50 bpm (*p* < 0.001). For the recovery phase, the absolute duration was 0.79 ± 0.31 s at 30 bpm and 0.60 ± 0.20 s at 50 bpm, significantly shorter at 50 bpm (*p* < 0.001). Conversely, the recovery-phase proportion of the cycle was 37.90 ± 11.89% at 30 bpm and 45.82 ± 5.51% at 50 bpm, which was significantly greater at 50 bpm (*p* < 0.01).

### 3.2. Muscle Activation

A comparative analysis of muscle activity was conducted under two propulsion conditions. A comparative analysis of EMG at four key instants during the propulsion cycle is summarized in Table 3. Most posterior arm position (prepare). Significant between-condition differences were observed for anterior deltoid (AD), pectoralis major (PM), biceps brachii (BB), and upper trapezius (UT). AD was 229.36 ± 76.63% RVC at 30 bpm and 517.78 ± 554.14% RVC at 50 bpm (*p* < 0.01); PM was 149.80 ± 48.23% RVC at 30 bpm and 178.58 ± 54.91% RVC at 50 bpm (*p* < 0.05); BB was 292.16 ± 179.76% RVC at 30 bpm and 423.02 ± 214.41% RVC at 50 bpm (*p* < 0.05); UT was 245.88 ± 151.47% RVC at 30 bpm and 333.54 ± 249.92% RVC at 50 bpm (*p* < 0.05). MD tended to be higher at 30 bpm, whereas PD and TB tended to be higher at 50 bpm; these trends were not significant.

Start (initial hand–rim contact). A significant difference was found only for PD: 257.08 ± 211.72% RVC at 30 bpm vs. 330.97 ± 268.04% RVC at 50 bpm (*p* < 0.05). For the other muscles, BB and UT were relatively higher at 30 bpm, whereas AD, MD, PM, and TB were relatively higher at 50 bpm; none reached significance.

End (hand–rim release). A significant difference was found only for BB: 572.62 ± 545.61% RVC at 30 bpm vs. 228.59 ± 139.44% RVC at 50 bpm (*p* < 0.01). For the remaining muscles, PM and PD were relatively higher at 30 bpm, whereas AD, MD, TB, and UT were relatively higher at 50 bpm; differences were not significant.

Finish (maximal forward reach after push). A significant difference was observed only for AD: 852.60 ± 593.16% RVC at 30 bpm vs. 475.40 ± 676.37% RVC at 50 bpm (*p* < 0.05). For the other muscles, PD, PM, and BB tended to be higher at 30 bpm, whereas MD, TB, and UT tended to be higher at 50 bpm; none were significant. To compare muscle activity by phase, integrated EMG (iEMG) was computed; the results are presented in Table 4. During the push phase (hand in contact with the handrim), significant differences were observed for the pectoralis major (PM) and biceps brachii (BB). PM was 303.07 ± 225.91% RVC·s at 30 bpm and 365.60 ± 242.24% RVC·s at 50 bpm, being significantly greater at 50 bpm (*p* < 0.01). BB was 417.57 ± 311.23% RVC·s at 30 bpm and 767.74 ± 823.39% RVC·s at 50 bpm, also significantly greater at 50 bpm (*p* < 0.01). For the remaining muscles, AD, TB, and UT exhibited higher iEMG at 30 bpm, whereas MD and PD showed higher values at 50 bpm; however, these trends were not statistically significant. During the recovery phase (hand off the handrim while the arm returns for the next push), a significant difference emerged only for BB: 422.99 ± 1054.51% RVC·s at 30 bpm vs. 296.60 ± 717.86% RVC·s at 50 bpm, being significantly greater at 30 bpm (*p* < 0.05). For the other muscles, AD and BB presented higher iEMG at 30 bpm, whereas MD, PD, TB, and UT showed higher values at 50 bpm; again, no significant differences were observed.

## 4. Discussion

With the growing number of manual wheelchair users, the need for efficient mobility to sustain independence and quality of life has heightened the importance of improving manual wheelchair propulsion performance [15]. To develop effective rehabilitation programs that promote long-term health and functional independence in individuals with SCI, understanding optimal propulsion strategies is essential [8]. Therefore, unlike previous studies that focused on different speeds, this study was able to identify manual wheelchair propulsion strategies according to two propulsion conditions (30 bpm vs. 50 bpm) and confirm the differences.

In this study, the ROM for shoulder flexion/extension, shoulder adduction/abduction, and elbow flexion/extension did not differ significantly between the 30 bpm and 50 bpm conditions. Previous studies have reported that shoulder kinematics change significantly as speed increases when comparing self-selected speed and target speed [16]. Additionally, it was reported that the ROM of the shoulder, elbow, and wrist tended to increase with the slope and curb height [17]. In this study, although there was no significant difference between the two propulsion conditions, a tendency to decrease was confirmed at 50 bpm, and the decrease was substantial in the elbow joint during the recovery phase. This is thought to be because the ROM of the upper extremity joints did not show a significant difference. The two propulsion conditions with a constant rhythm were limited when propelling a manual wheelchair, and timing control was prioritized over the form of joint movement when propelling a manual wheelchair at a continuous rhythm.

In this study, the push angle was, on average, larger at 50 bpm, but the difference did not reach statistical significance. This contrasts with prior work, in which postural adjustments and wheelchair setup were found to exert apparent effects on push angle [18]. While modifications to wheelchair configuration/fit generally tend to increase push angle, task constraints such as speed or cadence may not produce consistent changes. In line with recent intervention findings that training and augmented feedback can increase the contact (push) angle and adjust push frequency [19], our results suggest that cadence manipulation alone, without concurrent technique coaching or configuration changes, may be insufficient to elicit a significant alteration in push angle. Under the present iso-velocity, metronome-constrained design, participants likely accommodated the higher cadence primarily via temporal rather than spatial adjustments, limiting angle modulation.

In the temporal domain of the propulsion cycle, cycle duration was significantly shorter at 50 bpm. Although the absolute duration of the push phase did not differ significantly between conditions, its proportion of the cycle was significantly greater at 50 bpm. Prior studies report mixed findings: with increasing speed, the contact proportion has been shown to decrease while the recovery proportion increases; conversely, with greater incline or task-specific constraints, the contact proportion increases, indicating that outcomes depend on speed, incline, task, and, critically, on event definitions [17,20,21,22]. Since the design that controlled a constant rhythm, like this study, is rare, it is thought that the participants adjusted the timing of the recovery and preparation sections in accordance with the increase in rhythm while maintaining the time of the push phase.

Across the four event–based EMG comparisons, a consistent pattern emerged. At prepare (the most posterior arm position), the AD, PM, BB, and UT exhibited significantly greater activity at 50 bpm. This likely reflects anticipatory (feed-forward) recruitment of the scapular and anterior shoulder chain to establish hand–rim coupling at the higher cadence rapidly. Consistent with prior work, increases in task tempo/exigency are associated with larger EMG amplitudes, and co-activation during the preparatory interval appears to facilitate precise hand positioning and glenohumeral stability in faster movements [23].

At the start (initial hand–rim contact), the PD was significantly higher at 50 bpm. This likely reflects stronger recruitment of shoulder horizontal extension (i.e., transverse-plane backward motion of the humerus) to deliver initial propulsion torque more rapidly at the higher cadence. Prior reports indicate that, at the instant of hand contact, the shoulder is extended while the elbow is flexed, consistent with a coupled shoulder elbow action at push initiation [16,24,25]. Moreover, the initial contact angle has been linked to shoulder loading, underscoring the mechanical importance of shoulder posture at contact [17]. At the end (hand–rim release), BB was greater at 30 bpm. This suggests that, at the lower cadence, BB is recruited more for elbow-flexion braking and deceleration control as the push terminates, likely supporting a more leisurely transition into recovery. In contrast, at 50 bpm, the compressed recovery may reduce the need for late-phase braking or redistribute this demand to other muscles (e.g., triceps or scapular stabilizers). Recent literature similarly reports shifts in muscle-activation distribution with changes in speed and technique [23]. At the finish (maximal forward reach after push), AD was greater at 30 bpm. A lower cadence affords more time to prepare the next stroke, enabling a longer forward reach; conversely, the higher cadence may constrain anterior reach as the limb cycles more quickly toward the subsequent contact.

The relatively large standard deviations observed in the EMG data indicate substantial between-subject variability in muscle activation. This is not unexpected in individuals with spinal cord injury, who differ in lesion level and completeness, chronicity, and wheelchair propulsion experience, and who may rely on diverse compensatory movement strategies. In addition, EMG amplitudes were normalized to a reference voluntary contraction (RVC), and variability in the quality and magnitude of this reference contraction across participants may have further amplified between-subject differences in normalized (%RVC) values.

In summary, the results of this study suggest that increased propulsive rhythm appears to be driven by a tendency to adjust the magnitude and degree of muscle activation to match the rhythm, rather than by large-angle changes. Specifically, activation of agonist and stabilizing muscles during the initial contact phase of the propulsive cycle was prominent. This finding supports the validity of previous studies, which have reported increases in trunk and scapular agonist muscle activity during speed increases in racing athletes. However, caution is warranted in generalizing due to differences in subjects and experimental interventions.

Clinically, the observed differences in muscle activation suggest that higher cadence places greater demand on the anterior shoulder complex and scapular stabilizers during the preparatory and early push phases, potentially increasing shoulder loading in susceptible individuals. In contrast, the lower cadence condition appears to emphasize braking and controlled forward reach, with greater involvement of the biceps brachii and anterior deltoid at the end of the push and during the transition into recovery. These patterns indicate that cadence manipulation can be used to selectively train rapid preparatory activation versus controlled deceleration, and that cadence prescriptions should be individualized while monitoring shoulder symptoms and shoulder muscle capacity in manual wheelchair users. And from a clinical perspective, these findings suggest that cadence-focused wheelchair propulsion training may be used to modify temporal and neuromuscular aspects of propulsion without requiring large changes in joint range of motion or push angle. Metronome-guided practice at moderately higher cadences (50 bpm), combined with strengthening and endurance training of key agonist and stabilizing muscles, could be incorporated into wheelchair skills programs to improve preparatory activation at hand–rim contact, optimize push–recovery timing, and help manual wheelchair users achieve safe and efficient propulsion in daily life.

This study has several limitations. First, to ensure experimental consistency, testing was conducted in a laboratory setting on a fixed wheelchair roller ergometer, rather than on the ground. Compared with overground propulsion, roller ergometer propulsion does not require steering, obstacle avoidance, or continuous adjustments to surface conditions, and provides a largely constant resistance with limited visual and vestibular flow. As a result, trunk and shoulder demands related to balance control and directional changes are likely reduced, and temporal and kinematic variability may be lower than during real-world propulsion. These differences may have influenced joint motion and muscle activation patterns and should be considered when generalizing our findings to everyday wheelchair use. Because participants were propelled in an environment that differs from their usual real-world conditions, future work should evaluate propulsion in everyday environments and on routes frequently traversed by manual wheelchair users to enhance ecological validity. Second, our analyses focused on kinematics and EMG; we did not include dynamic variables directly related to propulsion (e.g., handrim force/torque, joint moments, shoulder contact forces). Regarding fatigue, each propulsion trial was of short duration with 1 min rest intervals between trials, and the overall workload at 50 bpm was modest. Participants did not report relevant shoulder fatigue or discomfort, and we did not observe a systematic drift in kinematic or EMG patterns across repetitions, suggesting that acute fatigue is unlikely to have substantially affected the main results. Nevertheless, the possibility of subtle fatigue-related effects cannot be completely excluded and should be examined in future studies using longer or repeated high-cadence propulsion bouts. Given emerging model-based approaches that estimate the effects of fatigue and environmental stressors on shoulder loading, integrating fatigue manipulations and prolonged propulsion protocols may substantially strengthen the clinical relevance of future studies. Concurrent assessment of peak handrim force/torque during the push phase would allow a more concrete and objective interpretation of any push-angle differences. Finally, cadence was constrained using a metronome, which may have altered participants’ natural propulsion patterns. The imposed rhythmic pacing likely encouraged more regular, cycle-to-cycle timing and may have led participants to prioritize matching the beat over selecting their spontaneously preferred combination of push frequency and push angle. In some individuals, this constraint could have suppressed natural temporal and spatial variability, while in others it may have induced compensatory strategies (shortening recovery or modifying muscle recruitment) to remain synchronized with the metronome. Consequently, the propulsion patterns observed under metronome-constrained conditions may differ from those adopted during self-selected, unconstrained propulsion, and the generalizability of our findings to habitual daily propulsion should be interpreted with caution. Subsequent studies should directly compare natural (self-selected speed) propulsion and metronome-constrained propulsion to quantify any strategy shifts attributable to rhythm enforcement.

## 5. Conclusions

This study identified differences in upper-limb kinematics and muscle activation between 30 bpm and 50 bpm cadence conditions during manual wheelchair propulsion. By jointly analyzing ROM, push angle, temporal variables, and muscle activity at key time events across the propulsion cycle, the findings provide foundational evidence for how propulsion rhythm itself can reorganize strategy and redistribute load. This approach can help translate biomechanical insights into actionable guidance for propulsion skills training and device design. Moreover, elucidating the cadence-dependent propulsion mechanism offers objective evidence to support the development and engineering of efficient, low-injury-risk propulsion strategies and alternative methods for manual wheelchair use.

## Figures and Tables

**Table 1:** General characteristics of participants.

	Participants (n = 18)
Age (y)	60.89 ± 8.92
Height (cm)	168.86 ± 7.10
Weight (kg)	66.64 ± 17.12
BMI (kg/m^2^)	23.07 ± 4.26
Body fat (%)	27.28 ± 6.70
Onset (Y)	18.06 ± 8.70
Dominant side	Left: 2/Right: 16
Lesion level	T: 14/L: 4
ASIA scale	A: 12/B: 0/C: 0/D: 6

BMI: kg/m^2^, T: Thorax, L: Lumbar, ASIA: American Spinal Injury Association.

**Table 2:** Kinematic comparative analysis results.

Variables	Joint (Axis)	30 bpm	50 bpm	Z	*p*
Propulsion cycle (deg)	Elbow (X)	50.99 ± 9.78	49.53 ± 7.50	−1.154	0.248
	Shoulder (X)	63.48 ± 11.45	62.60 ± 11.62	−1.023	0.306
	Shoulder (Y)	16.94 ± 5.89	15.86 ± 5.58	−1.633	0.102
Push phase (deg)	Elbow (X)	22.90 ± 9.57	22.90 ± 9.93	−0.022	0.983
	Shoulder (X)	33.65 ± 9.28	35.06 ± 9.07	−1.372	0.170
	Shoulder (Y)	6.22 ± 2.57	5.50 ± 1.99	−0.936	0.349
Recovery phase (deg)	Elbow (X)	60.45 ± 28.65	50.20 ± 14.00	−1.894	0.058
	Shoulder (X)	62.26 ± 11.66	61.47 ± 11.63	−0.893	0.372
	Shoulder (Y)	14.84 ± 6.43	13.65 ± 5.97	−1.764	0.078
Push angle (deg)	start	110.53 ± 12.29	111.94 ± 11.63	−1.023	0.306
	end	68.19 ± 6.15	68.04 ± 7.65	−0.022	0.983
	Push angle	42.34 ± 9.19	43.906.53	−1.241	0.215
Temporal parameters	propulsion cycle (s)	2.07 ± 0.25	1.29 ± 0.37	−3.724	<0.001 ***
	push phase (s)	0.33 ± 0.07	0.32 ± 0.10	−0.762	0.446
	push phase (%)	15.93 ± 2.18	24.93 ± 3.05	−3.724	<0.001 ***
	recovery phase (s)	0.79 ± 0.31	0.60 ± 0.20	−3.724	<0.001 ***
	recovery phase (%)	37.90 ± 11.89	45.82 ± 5.51	−2.722	0.006 **
	Cadence (propulsion/min)	29.46 ± 2.33	48.30 ± 6.86	−3.724	<0.001 ***

** *p* < 0.01; *** *p* < 0.001; X-axis: flexion/extension, Y-axis: abduction/adduction.

**Table 3:** Muscle activation comparative analysis results.

Event	Muscle	30 bpm	50 bpm	Z	*p*
prepare	AD	229.36 ± 76.63	517.78 ± 554.14	−3.245	0.001 **
	MD	462.63 ± 290.08	451.35 ± 471.31	−0.065	0.948
	PD	364.59 ± 279.50	373.43 ± 328.12	−0.501	0.616
	PM	149.80 ± 48.23	178.58 ± 54.91	−2.373	0.018 *
	BB	292.16 ± 179.76	423.02 ± 214.41	−2.417	0.016 *
	TB	268.11 ± 154.09	320.52 ± 177.71	−1.415	0.157
	UT	245.88 ± 151.47	333.54 ± 249.92	−2.199	0.028 *
start	AD	395.21 ± 282.88	564.57 ± 1081.76	−0.022	0.983
	MD	299.25 ± 228.69	308.88 ± 244.73	−0.588	0.557
	PD	257.08 ± 211.72	330.97 ± 268.04	−2.199	0.028 *
	PM	397.16 ± 264.15	463.48 ± 272.00	−0.893	0.372
	BB	366.19 ± 229.50	298.41 ± 205.28	−0.762	0.446
	TB	158.21 ± 58.15	165.28 ± 68.02	−0.675	0.500
	UT	365.75 ± 262.65	296.71 ± 199.52	−0.240	0.811
end	AD	352.63 ± 227.74	579.72 ± 567.37	−1.328	0.184
	MD	343.00 ± 187.36	604.12 ± 776.72	−1.154	0.248
	PD	337.41 ± 272.43	295.68 ± 235.38	−0.806	0.420
	PM	486.34 ± 267.73	432.68 ± 313.35	−0.022	0.983
	BB	572.62 ± 545.61	228.59 ± 139.44	−2.853	0.004 **
	TB	376.03 ± 207.98	387.85 ± 267.34	0.283	0.777
	UT	239.12 ± 110.08	259.65 ± 152.52	−0.283	0.777
finish	AD	852.60 ± 593.16	475.40 ± 676.37	−2.025	0.043 *
	MD	439.69 ± 216.26	460.59 ± 658.49	−0.936	0.349
	PD	398.51 ± 296.46	304.85 ± 199.19	−1.067	0.286
	PM	274.75 ± 181.02	239.80 ± 125.33	−1.241	0.215
	BB	457.06 ± 657.88	328.21 ± 208.60	−0.762	0.446
	TB	388.10 ± 218.65	399.98 ± 254.70	−0.762	0.446
	UT	347.92 ± 222.42	382.52 ± 265.31	−0.240	0.811

Unit: %RVC, * *p* < 0.05, ** *p* < 0.01, AD: anterior deltoid, MD: middle deltoid, PD: posterior deltoid, PM: pectoralis major, BB: biceps brachii, TB: triceps brachii, UT: upper trapezius.

**Table 4:** iEMG comparative analysis results.

Phase	Muscle	30 bpm	50 bpm	Z	*p*
push	AD	427.21 ± 269.53	307.81 ± 242.17	−1.154	0.248
	MD	118.24 ± 88.36	133.53 ± 109.85	−1.372	0.170
	PD	121.89 ± 107.13	124.26 ± 145.11	−0.719	0.472
	PM	303.07 ± 225.91	365.50 ± 242.24	−2.983	0.003 **
	BB	417.57 ± 311.23	767.74 ± 823.39	−2.635	0.008 **
	TB	110.83 ± 78.50	109.70 ± 58.42	−0.414	0.679
	UT	93.17 ± 55.28	93.08 ± 50.78	−0.544	0.586
follow	AD	300.12 ± 241.51	206.80 ± 145.89	−1.145	0.157
	MD	227.00 ± 162.57	254.15 ± 187.58	−1.415	0.157
	PD	231.68 ± 183.10	273.43 ± 240.40	−0.370	0.711
	PM	97.58 ± 85.88	114.88 ± 113.25	−0.936	0.349
	BB	422.99 ± 1054.51	296.60 ± 717.86	−2.330	0.020 *
	TB	244.90 ± 207.60	252.93 ± 280.80	−0.675	0.500
	UT	83.62 ± 50.83	86.65 ± 57.85	−0.414	0.679

Unit: %RVC, * *p* < 0.05, ** *p* < 0.01, AD: anterior deltoid, MD: middle deltoid, PD: posterior deltoid, PM: pectoralis major, BB: biceps brachii, TB: triceps brachii, UT: upper trapezius.

## Data Availability

The data presented in this study are available on request from the corresponding authors due to privacy and ethical restrictions related to participant confidentiality.
